# Inhibition of UBA5 Expression and Induction of Autophagy in Breast Cancer Cells by Usenamine A

**DOI:** 10.3390/biom11091348

**Published:** 2021-09-11

**Authors:** Bo Fang, Zijun Li, Yinda Qiu, Namki Cho, Hee Min Yoo

**Affiliations:** 1 Research Institute of Pharmaceutical Sciences, College of Pharmacy, Chonnam National University, Gwangju 61186, Korea; fangboplato@163.com (B.F.); dalizijun1996@gmail.com (Z.L.); qiuyinda51@163.com (Y.Q.); 2 Biometrology Group, Korea Research Institute of Standards and Science (KRISS), Daejeon 34113, Korea; 3 Department of Bio-Analytical Science, University of Science & Technology (UST), Daejeon 34113, Korea

**Keywords:** usenamine A, UBA5, breast cancer, ER stress, autophagy

## Abstract

Breast cancer is now the most common type of cancer worldwide, surpassing lung cancer. This issue is further worsened by the lack of effective therapies for the disease. Recent reports indicate that the inhibition of ubiquitin-like modifier-activating enzyme 5 (UBA5) can impede tumor development. However, there have been few reports regarding UBA5-inhibiting compounds. This work studied usenamine A, a natural product from the lichen *Usnea longissimi* that exhibits UBA5-inhibitory effects. Bioinformatics analysis was performed using public databases, and the anti-proliferative ability of usenamine A in breast cancer cells was examined through MTS and colony formation assays. Flow cytometry and western blot analysis were also conducted to examine and analyze cell cycle arrest and apoptosis. In addition, LC3B-RFP and UBA5 expression plasmids were used for the analysis of usenamine A-induced autophagy. According to the bioinformatics analysis results, UBA5 was upregulated in breast cancer. According to in vitro studies, usenamine A displayed prominent anti-proliferative activity and resulted in G2/M phase arrest in MDA-MB-231 cells. Moreover, usenamine A induced autophagy and endoplasmic reticulum stress in MDA-MB-231 cells. In conclusion, the findings support the potential of usenamine A as an agent that can attenuate the development and progression of breast cancer.

## 1. Introduction

In recent years, breast cancer has become prevalent to the point that it is now the most common form of cancer and the second most prevalent cause of cancer-related death in women [1]. According to the latest global cancer data published by the International Agency for Research on Cancer (IARC) of the World Health Organization, lung cancer has been replaced by breast cancer as the most commonly diagnosed cancer worldwide, with a global incidence of 2.26 million new cases in 2020. Over the past few decades, several subtypes of breast cancer have been classified based on the expression of estrogen receptors, progesterone receptors, and human epidermal growth factor receptor 2. Basal-like or triple-negative breast cancer (TNBC) is a subtype that lacks the expression of the aforementioned receptors and comprises ~15% of all invasive breast cancer cases [2]. Although atezolizumab, a monoclonal antibody and an immune checkpoint inhibitor, is currently approved for the treatment of TNBC, there remains a deficiency of precise and effective small-molecule drugs provided by health clinics for the treatment of TNBC.

Apoptosis (also known as type I cell death) and autophagy (also known as type II cell death) are two primary mechanisms of programmed cell death [3]. Apoptosis is characterized by morphological and structural changes, including plasma membrane blebbing, nucleus and chromatin condensation, and attenuation of mitochondrial membrane potential [4]. Autophagy is an important catabolic cell process in which damaged cytoplasmic proteins or organelles are degraded and recycled via delivery to lysosomes to maintain cellular homeostasis [5]. While autophagy primarily promotes cell survival, its sustained activation can induce autophagic or apoptotic cell death. Additionally, it is clear that apoptosis and autophagy can simultaneously occur in the same cells through various stress pathways [6].

Similar to ubiquitination, the UFMylation system promotes the conjugation of UFM1 to its target protein [7]. In general, the UFMylation system is activated following pharmacological ER stress and protects cells under abnormal conditions [8,9]. Several studies showed that the inhibition of the UFMylation system is a promising treatment against various tumors [10]. UBA5 is an E1-activating enzyme that plays an essential role in the UFMylation system. A previous study showed that the knockdown of UBA5 effectively prevents the growth of breast cancer [11]. Another study also indicated that the selective inhibition of UBA5 can hinder the development of pancreatic cancer in vitro and in vivo [12]. Although UBA5 is a promising target for cancer treatment, only two UBA5 inhibitors have been reported in the literature [12,13]. Therefore, we screened a series of natural products to identify a drug capable of inhibiting UBA5. Based on our research, usenamine A, a natural product from *Usnea longissimi*, was found to inhibit the expression of UBA5. However, the effect of usenamine A on breast cancer remains to be further studied.

Therefore, we aimed to study the growth inhibitory effects of usenamine A against breast cancer cells and identify the underlying mechanisms through which inhibition takes place. According to our research, UBA5 was overexpressed in a broad spectrum of tumors, including breast cancer. Furthermore, UBA5 was associated with poor prognosis. As a result of our study, it was determined that usenamine A promotes apoptosis and autophagy to inhibit the growth of MDA-MB-231 cancer cells. Furthermore, the results showed that overexpression of UBA5 reduced the autophagic flux in MDA-MB-231 cancer cells, which could be reversed by usenamine A. Through our research, we shed new light into the development of UBA5 inhibitors and their application in cancer treatment.

## 2. Materials and Methods

### 2.1. Bioinformatics Analysis

The Gene Expression Profiling Interactive Analysis (GEPIA) database [14,15] and UALCAN analysis tools [16,17] were used to assess the expression levels of UBA5 in various tumors. The correlation between UBA5 and overall survival rates in breast cancer was also assessed by the survival analysis module of GEPIA. Patients were divided into two groups using quartiles as the cut-off points, and median survival was computed.

### 2.2. Enrichment Analysis of Gene Ontology (GO)

The functional protein association network was analyzed using STRING [18,19], and GO analysis was performed using DAVID [20,21].

### 2.3. Cell Culture

The human Caucasian breast adenocarcinoma cell line MDA-MB-231 was purchased from the Korean Cell Line Bank (KCLB, Seoul, Korea). To grow MDA-MB-231 cells, the cells were placed in culture flasks containing RPMI-1640 (Gibco, Eggenstein, Germany) medium supplemented with 10% fetal bovine serum (FBS) and 1× antibiotic/antimycotic (Gibco, Eggenstein, Germany) and were cultured under a 5% CO_2_ atmosphere at 37 °C.

### 2.4. Cell Viability Assay

Cell proliferation of MDA-MB-231 cancer cells upon treatment with usenamine A was measured as follows. In total, 8 × 10^3^ cells were seeded into 96-well plates, incubated overnight, then exposed for 48 h to different concentrations of the developed drug. Then, 3-(4,5-di-methylthiazol-2-yl)-2,5-diphenyl-2 tetrazolium bromide (MTT) solution was added to each well, and the cells were incubated at 37 °C under 5% CO_2_ for 4 h. The culture medium was removed, and 100 μL DMSO was added to each well to dissolve formazan crystals. Cell viability was measured by reading the absorbance of the resulting solution at 490 nm using a spectrophotometer (DTX880, Beckman Coulter, CA, USA).

### 2.5. Microscopic Analysis

To analyze the changes in the morphology of the MDA-MB-231 cells, microscopic analysis was conducted using a phase-contrast microscope. Photomicrographs of the cells were taken 24 h and 48 h after treatment with usenamine A, and changes in the number of cells were analyzed.

### 2.6. Colony Formation Assay

MDA-MB-231 cells were seeded in 12-well adhesive culture plates at a density of 800 cells/well and allowed to attach overnight. The cells were cultured for 15 days at 37 °C in standard growth media and incubated with different concentrations of usenamine A (5, 10, and 20 μM) to assess colony growth. The colonies were washed twice with phosphate-buffered saline (PBS), fixed with 4% paraformaldehyde for 15 min, and stained with 0.04% crystal violet for 15 min. After washing twice with distilled water, the colonies were observed under a light microscope. The colonies were counted in three independent experiments [22].

### 2.7. Staining Assay Using 5-Ethynyl-2′-Deoxyuridine (EdU) 

MDA-MB-231 cells were seeded in 6-well plates for EdU staining. After treatment for 48 h, 50 μM EdU solution was added, and the cells were incubated for 2 h. The cells were then treated with an EdU staining proliferation kit (Beyotime, Shanghai, China) and observed using a Nikon Fluorescence Microscope.

### 2.8. Cell Cycle Distribution Analysis

As previously described, MDA-MB-231 cells were cultured in 6-well plates and treated with usenamine A. Following trypsinization, the cells were collected, washed with PBS, and fixed at 4 °C in 70% ethanol for 24 h. The cells were again washed with PBS, then stained with 50 μg/mL of PI (propidium iodide) and 100 μg/mL of RNase solution in PBS for 30 min at room temperature. The cell cycle distribution was subsequently assessed using a FACSalibur flow cytometer (BD Biosciences, Franklin Lakes, NJ, USA).

### 2.9. Cell Apoptosis Analysis

We used a FITC Annexin V Apoptosis Detection Kit (BD Biosciences, San Jose, CA, USA) for apoptosis analysis. The cells were seeded into 6-well culture plates for 24 h and treated with usenamine A for 48 h. The cells were then harvested, washed with PBS, and resuspended in 1× Binding Buffer. This was followed by the addition of 5 μL of propidium iodide and 5 μL of FITC Annexin V. After incubation for 15 min, 400 μL of 1× Binding Buffer was added to the tube, and flow cytometry (BD Biosciences, Franklin Lakes, NJ, USA) was performed to investigate the stained cells.

### 2.10. Transwell Assay

To analyze cell invasion, we utilized the Transwell^®^ Permeable Supports, Polycarbonate Membrane (Corning Incorporated, Corning, NY, USA). After subjecting the cells to treatment, the cells were prepared using serum-free medium to achieve a concentration of 1 × 10^6^/mL. The cells were plated in Matrigel (50 μL)-coated Transwell inserts. Subsequently, 600 μL of a medium with 10% FBS was added into the lower chamber. After 100 μL of the prepared cell suspension was pipetted from each group into the upper chamber, the 24-well plate was cultured in a 37 °C incubator. After 24 h, the fluid and cells in the chamber were discarded, and the cells were washed three times with pre-warmed PBS. The cells were fixed with 4% paraformaldehyde for 15 min at room temperature, and DD water was used to wash the cells on the chamber membrane for three times. Afterwards, crystal violet was used to stain the cells for 5 min. DD water was again used with 30% glacial acetic acid to rinse the chamber, which resulted in crystal violet dissolution. Finally, the solution absorbance was measured at 560 nm.

### 2.11. Plasmids

Plasmids were transfected using the polyethyleneimine (PEI) reagent. The expression of plasmids was allowed for 24 h. The plasmids used in this study are pCMV-Tag3-UBA5 and RFP-LC3B.

### 2.12. Western Blot

The cells were lysed and boiled for 10 min. Subsequently, proteins were separated by SDS-PAGE, then transferred to a PVDF membrane. After being blocked in 5% dry milk in TBST for 1.5 h and washed with 1× TBST, the PVDF membrane was incubated with the following primary antibodies, purchased from Santa Cruz Biotechnology (Dallas, TX, USA) or Cell Signaling Technologies (Danvers, MA, USA), against Bax, Cleaved-caspase-3, Bcl-2, Cyclin B1, Cdc2, Cyclin A, Bip, Ire1α, Perk, Chop, p62, LC3B, Beclin1, p62, Atg-7, UBA5, and GAPDH. The ECL western blotting detection reagents were purchased from Thermo Scientific, and further analysis was conducted in ChemiDoc MP (Bio-Rad, Hercules, CA, USA).

### 2.13. RFP-LC3B Puncta Assay

Autophagy analysis was conducted using cells transfected with RFP-LC3B by examining the formation of fluorescent puncta of autophagosomes. The cells were transfected with 2 μg/mL RFP-LC3B plasmid in 6-well plates according to the manufacturer’s protocol. After transfection for 24 h, the cells were treated under different conditions. Images were acquired using a fluorescence microscope.

### 2.14. Molecular Docking

ChemOffice (ChemOffice 15.0, Cambridge, MA, USA) was used to construct the 3D structure of usenamine A and perform energy minimization. The crystal structure of the UBA5–UFM1 complex (PDB ID:6h77) was downloaded from the Protein Data Bank [23,24,25]. Following a structural optimization process, a usenamine A molecule and UBA5 protein were prepared in pdbqt format using AutoDockTools [26]. The molecular docking program was carried out using Autodock4 (Autodock4, Scripps Research Institute, San Diego, USA) and visualized using PyMOL (PyMOL 2.4, New York, NY, USA) and LigPlot (LigPlot1.4, European Bioinformatics Institute, Cambridge, UK) [27]. The grid box size of the ATP binding pocket was set to 42 Å, 44 Å, and 44 Å for x, y, and z, respectively, and the grid center was set to 10.032 Å, 9.421 Å, and 78.333 Å for x, y, and z, respectively. In addition, the grid box size of the potential binding pocket between UFM1 and UBA5 was set to 86 Å, 84 Å, and 88 Å for x, y, and z, respectively, and the grid center was set to 24.488 Å, 14.346 Å, and 90.199 Å for x, y, and z, respectively. The Lamarckian genetic algorithm (LGA) was applied for docking calculations.

### 2.15. Isolation of Usenamine A

To isolate usenamine A, the ethyl acetate fraction from the aerial parts of *Usnea longissimi* was subjected to silica gel column chromatography and eluted with a CH_2_Cl_2_–MeOH solvent mixture (from 100:1 to 1:100; *v*/*v*), which yielded seven fractions (E1–E7). E3 was further resolved using RP C18-MPLC (20% methanol to 90% methanol). Usenamine A (purity > 98%, HPLC analysis, Supplementary Materials Figures S1–S5) was obtained after the separation of subfraction E3-7 using semipreparative high-performance liquid chromatography with 70% CH_3_CN in H_2_O.

### 2.16. Statistical Analysis

Data were analyzed using Student’s two-tailed unpaired *t*-test. The *p*-values are indicated in the figures (* *p* < 0.05; ** *p* < 0.01; *** *p* < 0.001).

## 3. Results

### 3.1. UBA5 Is Highly Expressed in Breast Cancer and Correlates with Poor Prognosis

**Figure 1:**
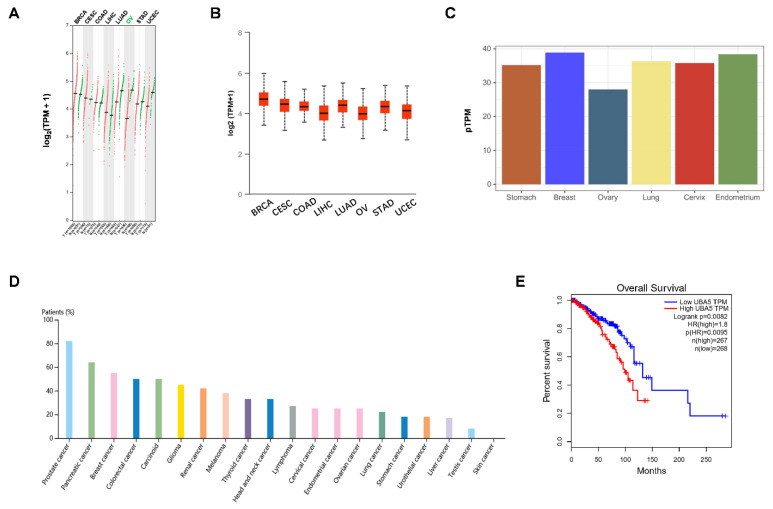
Validation of the expression levels and survival analysis of UBA5. (**A**,**B**) mRNA expression levels of UBA5 based on the GEPIA and UALCAN public databases, respectively. (**C**). UBA5 protein expression levels in various normal tissues, as obtained from the Human Protein Atlas database. (**D**) UBA5 protein expression in cancer patients based on the Human Protein Atlas database for each type of cancer. The color-coded bars indicate the percentage of patients (maximum 12 patients) with high and medium protein expression levels. (**E**). Overall survival analysis of UBA5 in breast cancer. TPM: transcripts per million.

The mRNA expression levels of UBA5 in different types of cancer was obtained from the GEPIA and UALCAN databases. As shown in Figure 1A, UBA5 was upregulated in several tumor types, including breast invasive carcinoma (BRCA), cervical squamous cell carcinoma (CESC), and colon adenocarcinoma (COAD). However, in other tumors, such as ovarian serous cystadenocarcinoma (OV), UBA5 was more highly expressed in normal samples than in tumor samples. Moreover, UBA5 was highly expressed in breast cancer than in other cancers, a result that was also confirmed by the UALCAN database (Figure 1B). To further verify the differential expression of UBA5, protein expression levels were evaluated using the Human Protein Atlas database. The result showed that UBA5 was more expressed in the breast than in other human tissues, such as the ovary (Figure 1C). Furthermore, as shown by the ranked percentages of different cancer patients according to the highest UBA5 expression in Figure 1D, UBA5 was overexpressed in breast cancer patients. Furthermore, survival analysis suggested that UBA5 was correlated with adverse prognosis (Figure 1E). Taken together, high expression of UBA5 was observed in breast cancer, and UBA5 expression may be a risk factor for breast cancer patients.

### 3.2. Construction of a PPI Network and GO Analysis

**Figure 2:**
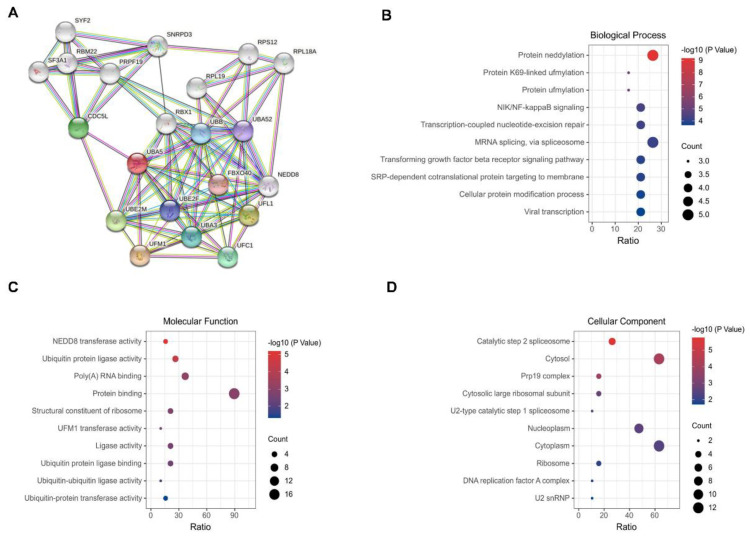
PPI network and GO analysis. (**A**) PPI network of UBA5. (**B**) Biological processes. (**C**) Cellular components. (**D**) Molecular functions. The size of the bubbles indicates the number of involved genes, and lighter colors represent smaller *p*-values.

GO enrichment analysis was performed to characterize the biological role of UBA5. With UBA5 as the core, a protein–protein interaction (PPI) network was initially constructed via STRING; here, a total of 21 main nodes were included (Figure 2A). For GO annotation, three aspects were considered: biological process (BP), cell composition (CC), and molecular function (MF). The 10 most significantly enriched GO terms were visualized and are shown in Figure 2B–D. The top 10 BP terms included protein NEDDylation, protein K69-linked UFMylation, protein UFMylation, NIK/NF-kappaB signaling, transcription-coupled nucleotide-excision repair, mRNA splicing, and transforming growth factor beta receptor signaling pathway (Figure 2B). Most of the top biological processes are related to post-translational modifications. In MF enrichment analysis, most MF entries were focused on ubiquitination, such as ubiquitin-protein ligase activity (Figure 2C). The top-ranked cellular component enrichment results consisted of catalytic step 2 spliceosome, cytosol, and Prp19 complex.

### 3.3. Usenamine A Inhibits the Proliferation and Invasion of MDA-MB-231 Cells

**Figure 3:**
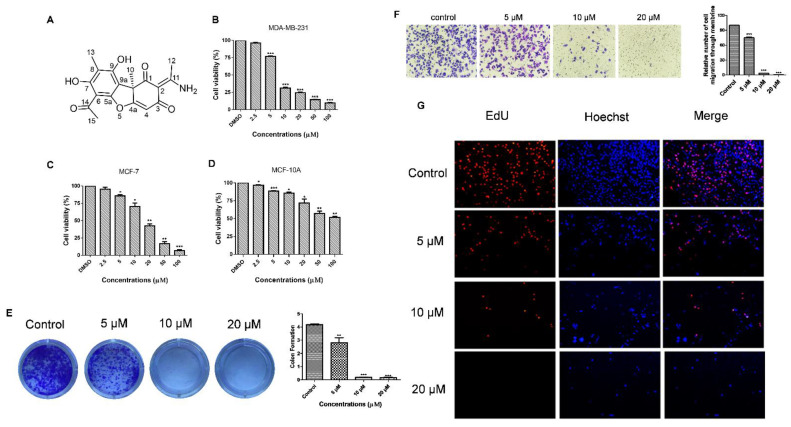
Anti-proliferation and anti-invasion capabilities of usenamine A. (**A**) Chemical structure of usenamine A. (**B**) Cell viability of MDA-MB-231 cells was evaluated through the MTT assay. Cells were treated with different concentrations of usenamine A for 48 h. The results are presented as relative cell viability (%). (**C**) Cell viability of MCF-7 cells was evaluated through the MTT assay. (**D**) Cytotoxicity of usenamine A against normal cells. (**E**) Inhibition of the colony-forming ability of MB-MD-231 cells by usenamine A, as detected by the clonogenic assay. The cells were cultured for 15 days at 37 °C in standard growth media and incubated with different concentrations of usenamine A (5, 10, and 20 μM) to assess colony growth. (**F**) Inhibition of the invasive capacity of MB-MD-231 cells by usenamine A as detected by the Transwell assay. (**G**) Number of 5-ethynyl-2′-deoxyuridine (EdU)-positive MDA-MB-231 cells after treatment with usenamine A for 48 h. Fetal bovine serum (FBS) was used as a supplement to culture MDA-MB-231 and MCF-7 cells. In the bar chart, the data represent mean ± standard deviation (n = 3). * *p* < 0.05, ** *p* < 0.01 and *** *p* < 0.001 compared to the control group.

To determine the cytotoxicity of usenamine A, MDA-MB-231 cells were subjected to usenamine A treatment at various concentrations, and cell viability was examined via the MTT assay. The structure of usenamine A is shown in Figure 3A. The MTT results showed that usenamine A is capable of reducing the cell viability of both basal-like breast cancer (MDA-MB-231 and Hs578T) and luminal breast cancer (MCF-7) cells dose-dependently (Figure 3B,C and Supplementary Materials Figure S6). More importantly, usenamine A possesses lower cytotoxicity against normal breast cells (MCF-10A, Figure 3D). The results of the colony formation assay showed that a reduced number of colonies was observed in the drug-treated group (Figure 3E), whereas almost no colonies were formed at a concentration of 20 μM. Additionally, the EdU assay was performed to further verify the effect of usenamine A on breast cancer cell proliferation. As shown in Figure 3G, usenamine A significantly decreased the number of EdU-positive cells. There were few EdU-positive cells observed at concentrations of 10 and 20 μM. Given that metastasis is the major cause of death among breast cancer patients [28], the effects of usenamine A on MDA-MB-231 cell invasion was examined through the Transwell assay. As a result, it was found that usenamine A dose-dependently decreased the invasion of breast cancer cells through the Transwell filter. After treatment with usenamine A at 10 μM and 20 μM concentrations, only 3.6% and 0.45% of invading cells were observed, respectively (Figure 3F). According to these findings, it can be said that usenamine A effectively inhibits the malignant proliferation and invasion of breast cancer cells.

### 3.4. Usenamine A Induces Cell Apoptosis and G2/M Phase Arrest in MDA-MB-231 Cells

**Figure 4:**
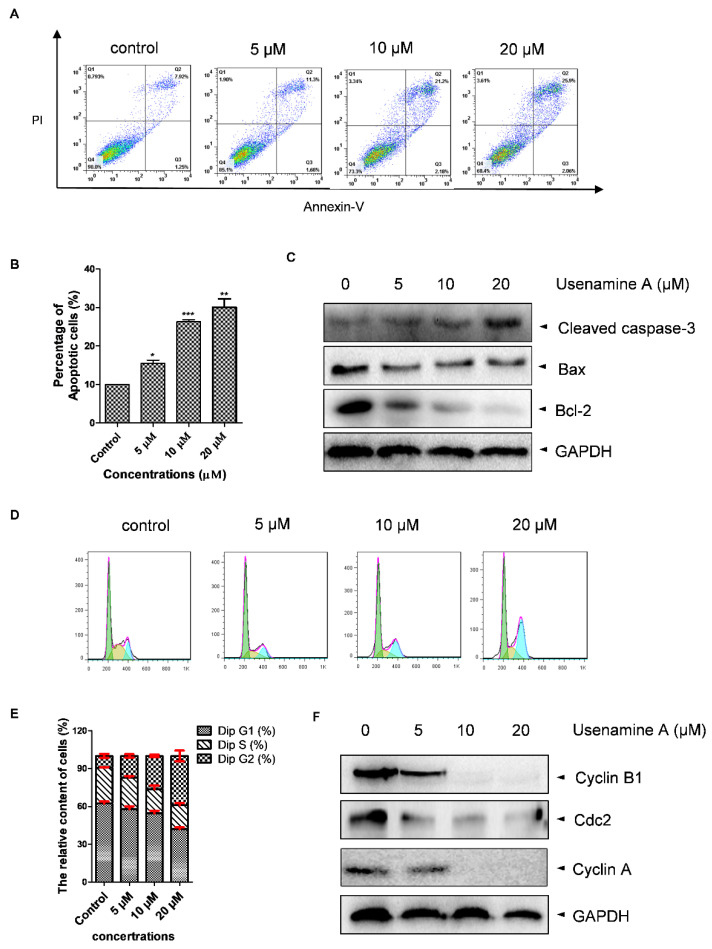
Apoptosis and cell cycle distribution analyses of MDA-MB-231 cells treated with usenamine A. (**A**,**B**) Apoptosis as determined by flow cytometry analysis following treatment with usenamine A (0, 5, 10, 20 μM) for 48 h. The apoptosis rate is the sum of the early and late apoptosis rates. (**C**) Representative western blot of apoptosis-related proteins. Data are representative of three independent experiments or are presented as the mean ± SD of three independent experiments. (**D**,**E**) Cell cycle distribution as examined by flow cytometry analysis following treatment with usenamine A (0, 5, 10, 20 μM) for 48 h. (**F**) Representative western blot of G2/M-related proteins. Fetal bovine serum (FBS) was used as a supplement to culture MDA-MB-231 cells. Values indicate mean ± SD of three independent experiments. * *p* < 0.05, ** *p* < 0.01 and *** *p* < 0.001 compared to the control group.

Apoptosis and cell cycle distribution were determined via flow cytometry and western blotting analysis. As shown in Figure 4A,B, cell apoptosis was dose-dependently induced by usenamine A. Consistent with the flow cytometry results, the western blotting assay showed that usenamine A treatment resulted in increased expression levels of cleaved-caspase-3 and decreased expression levels of Bcl-2 (Figure 4C). However, the change in Bax was not significant. Additionally, cell cycle analysis revealed that usenamine A induced MDA-MB-231 cells to undergo cell cycle arrest at the G2/M phase in a dose-dependent manner (Figure 4D,E). Furthermore, the western blotting assay showed that usenamine A treatment dose-dependently decreased the expression of Cyclin B1, Cdc2, and Cyclin A (Figure 4F). Taken together, these results demonstrate that usenamine A induces apoptosis and G2/M phase arrest in MDA-MB-231 cells.

### 3.5. Usenamine A Activates ER Stress and Induces Autophagy in MDA-MB-231 Cells

**Figure 5:**
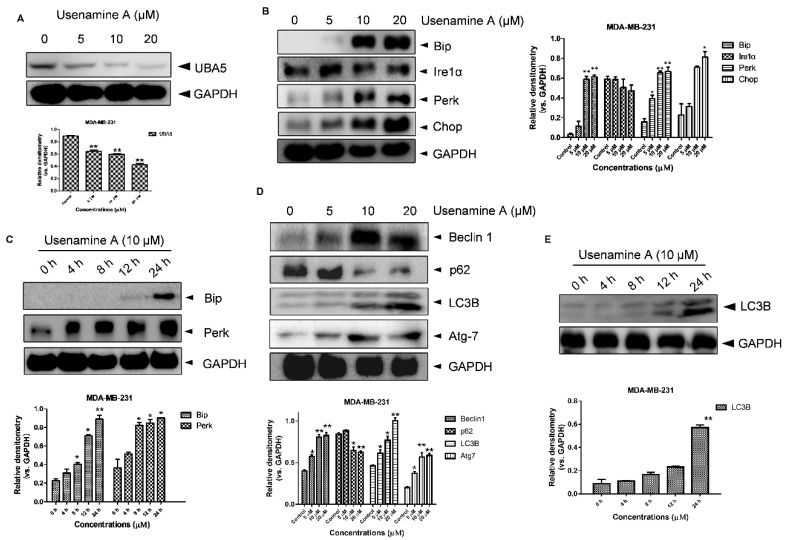
Usenamine A-induced ER stress and autophagy in MDA-MB-231 cancer cells. (**A**) Western blotting analysis of UBA5 expression in MDA-MB-231 cells. The cells were incubated with different concentrations of usenamine A (5, 10, and 20 μM) for 48 h. (**B**) Western blotting analysis of ER stress-related proteins in MDA-MB-231 cells. The cells were cultured for 48 h in standard growth medium and incubated with different concentrations of usenamine A (5, 10, and 20 μM). (**C**) Western blotting analysis of ER stress-related proteins in MDA-MB-231 cells. The cells were cultured in standard growth medium and incubated with the developed drug (10 μM) for various times (0, 4, 8, 12, 24 h). (**D**) Western blotting analysis of autophagy-related proteins in MDA-MB-231 cells. The cells were incubated with different concentrations of usenamine A (5, 10, and 20 μM) for 48 h. (**E**) Representative western blot of autophagy-related proteins in MDA-MB-231 cells. The cells were cultured in standard growth medium and incubated with the developed drug (10 μM) for various times (0, 4, 8, 12, 24 h). Fetal bovine serum (FBS) was used as a supplement to culture MDA-MB-231 cells. * *p* < 0.05, ** *p* < 0.01 and *** *p* < 0.001 compared to the control group.

Several reports have indicated that UFMylation is involved in endoplasmic reticulum (ER) stress [29,30,31], the activation of which can induce apoptosis and autophagy [32,33]. We further explored whether usenamine A is able to activate ER stress and induce autophagy, considering it dose-dependently inhibited UBA5 expression in breast cancer cells (Figure 5A). As shown in Figure 5B, usenamine A treatment significantly increased the expression of Bip, Perk, and Chop in a dose-dependent manner, whereas the expression of Ire1α did not exhibit significant changes. Additionally, MDA-MB-231 cells were treated with usenamine A at a concentration of 10 μM, and the expression levels of Bip and Perk were validated at five time points (0, 4, 8, 12, 24 h). As shown in Figure 5C, the expression of Bip abruptly increased at the 12 h time point. Furthermore, the expression of Perk also significantly increased after usenamine A treatment in a time-dependent manner. The autophagic markers LC3B, Beclin1, Atg7 and p62 were also examined via western blotting analysis. As shown in Figure 5D, usenamine A treatment induced LC3B, Atg7, and Beclin1 protein expression and inhibited p62 protein expression. Additionally, usenamine A treatment also time-dependently raised the expression level of LC3B (Figure 5E). Taken together, these results suggest that usenamine A can activate ER stress and induce autophagy in MDA-MB-231 cells.

### 3.6. Overexpression of UBA5 Can Reduce Autophagy in MDA-MB-231 Cells

**Figure 6:**
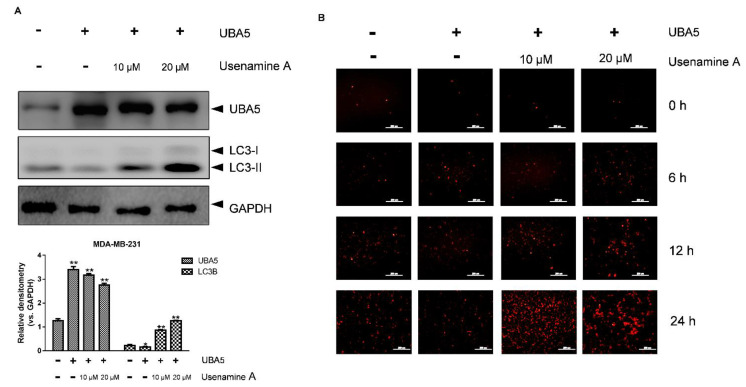
Overexpression of UBA5 inhibited autophagic activity in MDA-MB-231 cells. (**A**) Western blotting analysis of UBA5 and LC3B protein expression in MDA-MB-231 cells with or without the overexpression of UBA5. The cells were transfected with UBA5 for 24 h, rhen the medium was replaced with fresh growth medium, and the cells were treated with usenamine A (10 μM, 20 μM) or left untreated. (**B**) Immunofluorescence of RFP-LC3B (Red). The cells were transfected with UBA5 and RFP-LC3B for 24 h. After transfection, the cells were cultured in standard growth medium and treated with usenamine A (10 μM, 20 μM) or left untreated. Fetal bovine serum (FBS) was used as a supplement to culture MDA-MB-231 cells. * *p* < 0.05, ** *p* < 0.01 and *** *p* < 0.001 compared to the control group.

To further explore the role of UBA5 in autophagy induced by usenamine A, a UBA5 overexpression plasmid and the RFP-LC3B plasmid were constructed. MDA-MB-231 cells were transfected with the RFP-LC3B and UBA5 overexpression plasmids (UBA5+) or only the RFP-LC3B plasmid (UBA5-). After incubation for 24 h, drug treatment was performed (0, 10, 20 μM). As shown in Figure 6A, the western blot results indicated that the expression of UBA5 increased following transfection with the UBA5 overexpression plasmid. After overexpressing UBA5, cells treated with usenamine A did not exhibit significantly inhibition of UBA5 expression, compared to the untreated group. Usenamine A could only demonstrate slight UBA5 inhibitory activity at a concentration of 20 μM. Importantly, compared to the UBA5 group (no drug treatment), the expression of LC3B was decreased in the UBA5-overexpressing group (no drug treatment). However, increased expression levels of LC3B were observed again following usenamine A treatment, which indicates that usenamine A can reverse the inhibition of autophagy induced by the overexpression of UBA5. Similar results were obtained in the RFP-LC3B puncta-formation assay. As shown in Figure 6B, at the 24 h time point, attenuated LC3B-positive puncta were observed after overexpressing UBA5 in the untreated groups. Furthermore, cells treated with usenamine A exhibited an augmentation in LC3B-positive puncta. Taken together, these results indicate that overexpression of UBA5 inhibited the autophagic flux, which in turn was effectively reversed by usenamine A.

### 3.7. Molecular Docking of UBA5 to Usenamine A

**Figure 7:**
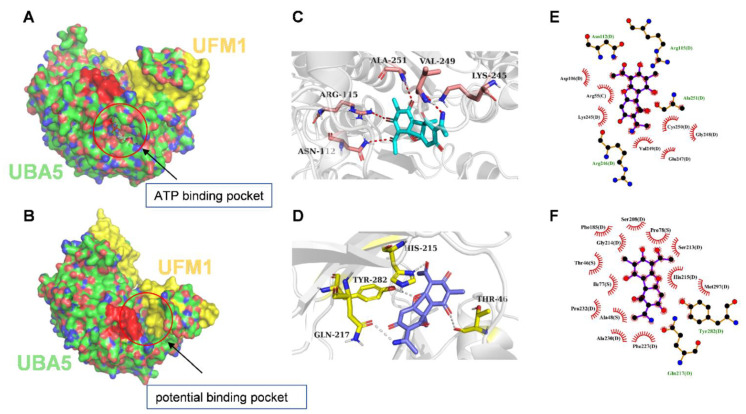
Calculated binding mode of usenamine A with a UBA5–UFM1 complex. (**A**) Overview of the ATP binding pocket. (**B**) Overview of the potential binding pocket. (**C**) Binding mode of usenamine A in the ATP binding pocket. (**D**) Binding mode of usenamine A in the potential binding pocket. (**E**) 2D representation of usenamine A in the ATP binding pocket. (**F**) 2D representation of usenamine A in the potential binding pocket.

To further explore the likelihood of usenamine A interacting with UBA5, molecular docking was performed. The crystal structure of the UBA5–UFM1 complex (6h77) was downloaded from the Protein Data Bank database. The structure of the UBA5–UFM1 complex showed that there were two possible binding pockets. One is considered as a classical ATP binding pocket (Figure 7A). and the other is a potential binding pocket (Figure 7B) between UFM1 and UBA5. In the figures, UFM1 and UBA5 are indicated in yellow and green, respectively. The molecular docking results showed that the docking score of usenamine A binding to the ATP pocket was only −5.43 kcal/mol, even if it was shown that several hydrogen bonds formed, including Asn112, Arg115, Ala251, Val249, and Lys245 (Figure 7C). Moreover, the 2D representation of usenamine A binding to the ATP pocket showed fewer hydrophobic interactions (Figure 7E). Therefore, it was worth further exploring the interface between UBA5 and UFM1. Figure 7D shows the best conformation of usenamine A in the potential binding pocket with a binding energy of −10.52 kcal/mol, which is significantly higher than the binding energy for the ATP binding pocket. In this case, the oxygen atoms at the C14 and C5 positions of usenamine A formed two strong hydrogen bonds with the His215 and Tyr282 residues of UBA5, respectively. Furthermore, the NH_2_ group at the C11 position was involved in hydrogen bonding with the oxygen atom of the main chain amino group of the Gln217 residue of UBA5, whereas the oxygen atom at the C9 position formed another hydrogen bond with Thr46 of UMF1. Moreover, as shown in Figure 7F, wide hydrophobic interactions formed. These data suggest that usenamine A has the potential to occupy the binding interfaces between UBA5 and UFM1.

## 4. Discussion

Various post-translational modifications are critical in regulating the functions of proteins [34,35]. In the case of ubiquitination, this process is widely involved in the post-translational modification of target proteins. Proteolysis Targeting Chimeras (PROTACs), which have garnered significant attention in recent years, exemplify the full use of the ubiquitination system in rational drug design [36]. The UFM1-activating enzyme (UBA5), UFM1-specific ligase (UFL1), UFM1-conjugating enzyme (UFC1), UFM1-binding protein (UFBP1), and UFM1-specific proteases (UFSP2) together constitute the UFMylation system, which is a kind of ubiquitination modification to which numerous human diseases are linked, including ischemic heart diseases, diabetes, atherosclerosis, hip dysplasia, schizophrenia, and cancer [37]. According to a study by Xi et al. on human osteosarcoma U2OS cells, cell proliferation and invasion is compromised by UFBP1 knockdown [38], and Jiang et al. reported the potential of UFM1 to covalently modify p53, stabilizing the latter [39]. These studies show that the regulation of critical components of UFMylation is a promising therapeutic strategy for cancer. Furthermore, two UBA5 inhibitors have been reported in recent years. Sara et al. designed a selective UBA5 inhibitor that incorporates an adenosine scaffold appended to a zinc (II) cyclen complex [13]. Allison et al. discovered a covalent ligand for UBA5 that impairs the growth of pancreatic cancer in vivo [12]. In a previous study, we reported the importance of UFMylation of ASC1 in the transactivation of ERα and breast cancer development [11]. However, the physiological functions and associated underlying molecular mechanisms of UBA5 in breast cancer remain to be explored.

Usenamine A, a natural product from the lichen *Usnea longissimi*, was found to inhibit the expression of UBA5 according to our up-front screening. In this study, we utilized the GEPIA and UALCAN databases to determine the expression of UBA5 in various cancers. We also discovered that UBA5 was upregulated in breast cancer. Further survival analysis indicated that high expression levels of UBA5 were related to poor prognosis in breast cancer patients. We subsequently performed a series of antitumor experiments using usenamine A due to its UBA5 inhibitory activity.

Apoptosis is a common mechanism by which natural products exert anti-cancer effects [40]. Cleaved-caspase 3 is one of the most important factors that induces apoptotic activity, whereas the apoptotic protein Bax and the anti-apoptotic protein Bcl-2 have antagonistic effects [41]. Through our research, we found that usenamine A promoted cell apoptosis in a dose-dependent manner, which was supported by our apoptosis analysis.

Many natural products have been reported to contribute to cell cycle arrest. Here, successful G2/M transformation is the key to cell division. In the early stages of the G2/M phase, the expression level of Cyclin B is initially low, and the peak expression level is achieved at the G2/M transition. The inactivation of Cyclin B–Cdc2 leads to G2/M phase arrest, and the G2/M phase transition is also promoted by Cyclin A [42]. In our study, we discovered that usenamine A treatment caused G2/M phase arrest in MDA-MB-231 cells. These phenotypes suggest that usenamine A promotes apoptosis by inducing cell cycle arrest at the G2/M phase in breast cancer cells.

Homeostasis of the endoplasmic reticulum (ER) is crucial to maintaining normal cellular functions [43]. Endoplasmic reticulum stress is a cellular response to homeostatic imbalances, which leads to a series of cell stress events such as the unfolded protein response (UPR). Recently, Ying et al. [43] proved that UPR is triggered by a knockdown of UFMylation system-related proteins in U2OS cells, which also results in the amplification of the ER network. Cai et al. [31] discovered that UFBP1 and other components of UFMylation can regulate the survival and differentiation of hematopoietic cells via modulation of ER homeostasis. Furthermore, the correlation between endoplasmic reticulum stress and UFMylation is becoming an increasingly important subject of research. Thus, we further explored whether usenamine A is able to activate ER stress. Through our research, we found that usenamine A treatment upregulated the expression of ER stress-related proteins, including Bip, Perk, and Chop. In general, the Bip protein is related to accumulated misfolded proteins under ER stress, which interferes with the interaction between Bip and Perk. Usenamine A enhanced the expression of Bip and Perk, which suggests that usenamine A is able to activate ER stress via the Perk pathway.

A disruption in endoplasmic reticulum (ER) homeostasis induces ER stress and leads to the unfolded protein response (UPR), which in turn leads to apoptosis [44]. The UBA5 E1 enzyme, which initiates UFM1 protein conjugation, is a critical enzyme in the UFM1 pathway [7]. A previous study reported that a deficiency of DDRGK domain-containing protein 1 (DDRGK1), a substrate adapter for the UFM1 pathway, leads to a significant accumulation of autophagosomes, promoting autophagy induction and ultimately aggravating apoptosis [45]. The mechanism of UBA5 stability is still unknown. One possible explanation is a negative feedback loop between the induction of ER stress, which increases autophagy, and autophagy-mediated UBA5 destabilization. Usenamine A inhibits UBA5 expression, thus activating ER stress and inducing autophagy. Therefore, an interesting topic for further study would be to determine whether UBA5 stability is regulated by autophagy.

To validate autophagic and apoptotic cell death by usenamine A, we performed an apoptosis assay after treatment with usenamine A and methyladenine (3-MA, an autophagy inhibitor). When co-treated with usenamine A and 3-MA, usenamine A-induced cell death was compromised (Supplementary Materials Figure S7). This explains the mechanism of apoptosis via the induction of autophagy.

A group of PI3K inhibitors, including 3-methyladenine (3-MA), are well known as autophagy inhibitors [46]. On the other hand, an accumulation of autophagic markers induced by prolonged 3-MA treatment is due to an increase in autophagic flux [47]. Previously, MDA-MB-231 cells exposed to 10 µM and 20 µM usenamine A showed increases in LC3B compared to the control sample (Figure 5D). Next, we performed an autophagy flux assay in MDA-MB-231 cells to verify the autophagic events. When co-treated with usenamine A and 3-MA, LC3B induction by usenamine A was compromised (Supplementary Materials Figure S8A). This explains the role of 3-MA as an autophagy inhibitor. Moreover, we performed an autophagy flux assay on MDA-MB-231 cells treated with bafilomycin A1 (BafA1), which inhibits autophagosome–lysosome fusion [48,49]. The autophagy flux assay revealed that usenamine A increased LC3 levels and autophagic flux. Moreover, co-treatment with usenamine A and BafA1 resulted in further accumulation of LC3-II (Supplementary Materials Figure S8B). Serum starvation is used as a positive control for autophagy studies. Autophagy can be activated in response to stress stimuli, including serum starvation, and LC3-II levels are known to increase in response to an absence of nutrients [50,51]. We performed an autophagy flux assay on MDA-MB-231 cells under serum starvation conditions. The autophagy flux assay revealed that usenamine A increased LC3 levels and autophagic flux. Simultaneous treatment with usenamine A and serum deprivation caused a further increase in LC3-II (Supplementary Materials Figure S8C). These data indicate that usenamine A with serum deprivation potentiated autophagy induction.

The natural self-cannibalization process of autophagy serves to remove aged and damaged organelles as well as protein aggregates [52]. There have been reports stating that autophagy is involved in the development of imatinib resistance of gastrointestinal stromal tumors [53]. However, prolonged activation of autophagy evokes autophagic programmed cell death [54]. Evidence has been provided regarding the strong potential of ER stress to induce autophagy [55]. Furthermore, autophagy also has a close connection with apoptosis, as it frequently precedes apoptosis [6]. As we identified that usenamine A induced apoptosis and activated ER stress in MDA-MB-231 cells, this led us to explore whether autophagy was activated by ER stress. Our results suggest that usenamine A treatment increased the expression of LC3B, which is indicative of increased autophagy. Moreover, overexpression of UBA5 could decrease the expression of LC3B in MDA-MB-231 cells, which in turn could be reversed by usenamine A. These findings suggest that UBA5 negatively regulates autophagy induction and that usenamine A is an efficient autophagy inducer.

## 5. Conclusions

In this study, we reported that usenamine A exhibits UBA5 inhibitory activity in breast cancer cells. Our results showed that UBA5 was upregulated in breast cancer and it expression was correlated with worse clinical outcomes. Additionally, we demonstrated that usenamine A decreased the expression of Cyclin B1 and Cyclin A in breast cancer cells, leading to cell cycle arrest at the G2/M phase. Moreover, usenamine A treatment induced breast cancer cells to undergo apoptosis and autophagy. Sustained ER stress was involved in the autophagic effect induced by usenamine A. Interestingly, overexpression of UBA5 inhibited the expression of LC3B, suggesting that UBA5 may be a suppressor of autophagy. However, the inhibition of LC3B induced by UBA5 could be reversed by usenamine A. Thus, usenamine A is a promising therapeutic agent for breast cancer, and further research efforts should focus on elucidating the mechanisms in detail.

## Data Availability

Data supporting the findings of this study are available from all first author on request.

## Supplementary Materials

The following are available online at https://www.mdpi.com/article/10.3390/biom11091348/s1, Figure S1: ^1^H NMR (400 MHz) spectrum of usenamine A in DMSO-*d*_6_, Figure S2. ^13^C NMR (100 MHz) spectrum of usenamine A in DMSO-*d*_6_, Figure S3. ^1^H-^1^H COSY spectrum of usenamine A in DMSO-*d*_6_, Figure S4. HSQC spectrum of usenamine A in DMSO-*d*_6_, Figure S5. HMBC spectrum of usenamine A in DMSO-*d*_6_, Figure S6. Effect of usenamine A on the viability of triple-negative breast cancer cells, Figure S7. Apoptosis analyses of MDA-MB-231 cells treated with usenamine A and 3-MA.

## Sample Availability

Samples of the compounds are available from the authors.
