# Supplementary material for: Inhibition of UBA5 Expression and Induction of Autophagy in Breast Cancer Cells by Usenamine A
Source: Biomolecules. 2021 Sep 11;11(9):1348. doi: 10.3390/biom11091348 (PMC8469757; doi:10.3390/biom11091348)
Supplement: Supplementary file 1 [file biomolecules-11-01348-s001.zip › biomolecules-1329093-supplementary.pdf]

biomolecules MDPI

Supporting Information

Inhibition of UBA5 Expression and Induction of Autophagy in Breast Cancer Cells by Usenamine A

Figure S1. ^{1}H NMR (400 MHz) spectrum of usenamine A in DMSO-d_6.

Figure S2. ^{13}C NMR (100 MHz) spectrum of usenamine A in DMSO-d_6.

Figure S3. ^{1}H-^{1}H COSY spectrum of usenamine A in DMSO-d_6.

Figure S4. HSQC spectrum of usenamine A in DMSO-d_6.

Figure S5. HMBC spectrum of usenamine A in DMSO-d_6.

Figure S6. Effect of usenamine A on the viability of triple-negative breast cancer cells.

Figure S7. Apoptosis analyses of MDA-MB-231 cells treated with usenamine A and 3-MA.

Figure S8. Representative images of immunoblot analysis results of LC3B obtained from autophagy flux assays under 3-MA, BafA1, or serum starvation.

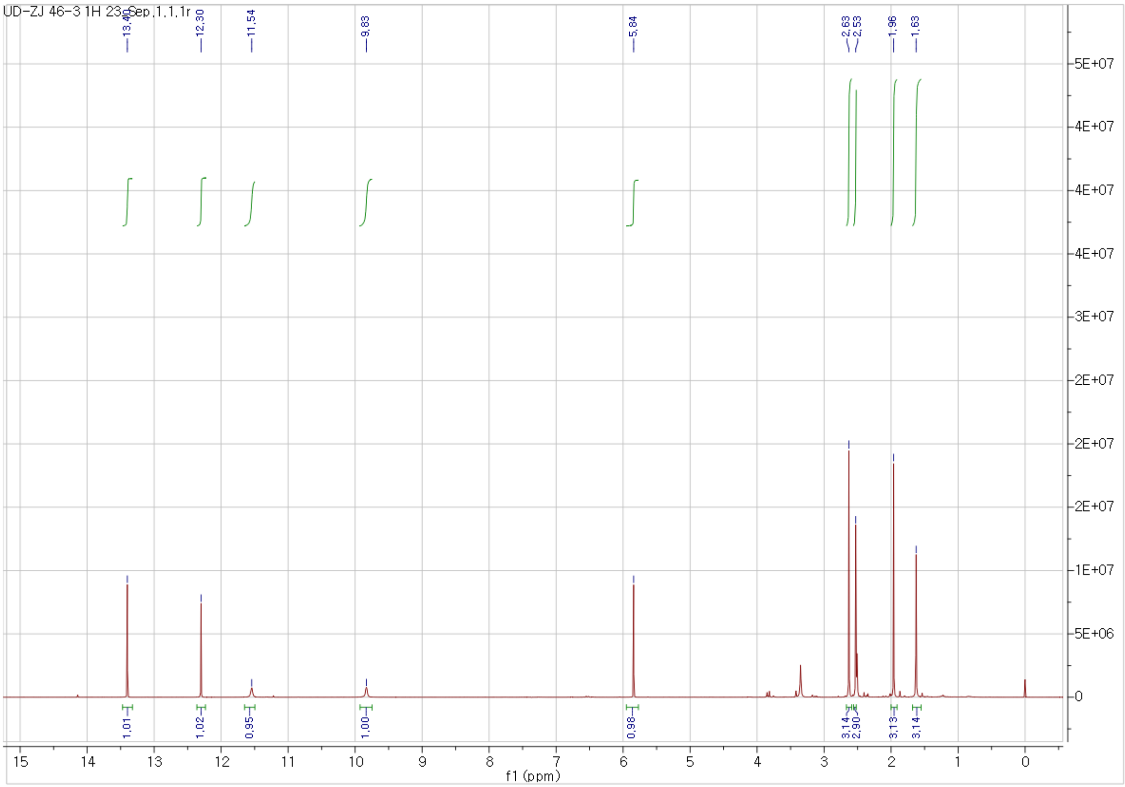

Figure S1. ^{1}H NMR (400 MHz) spectrum of usenamine A in DMSO-d_6.

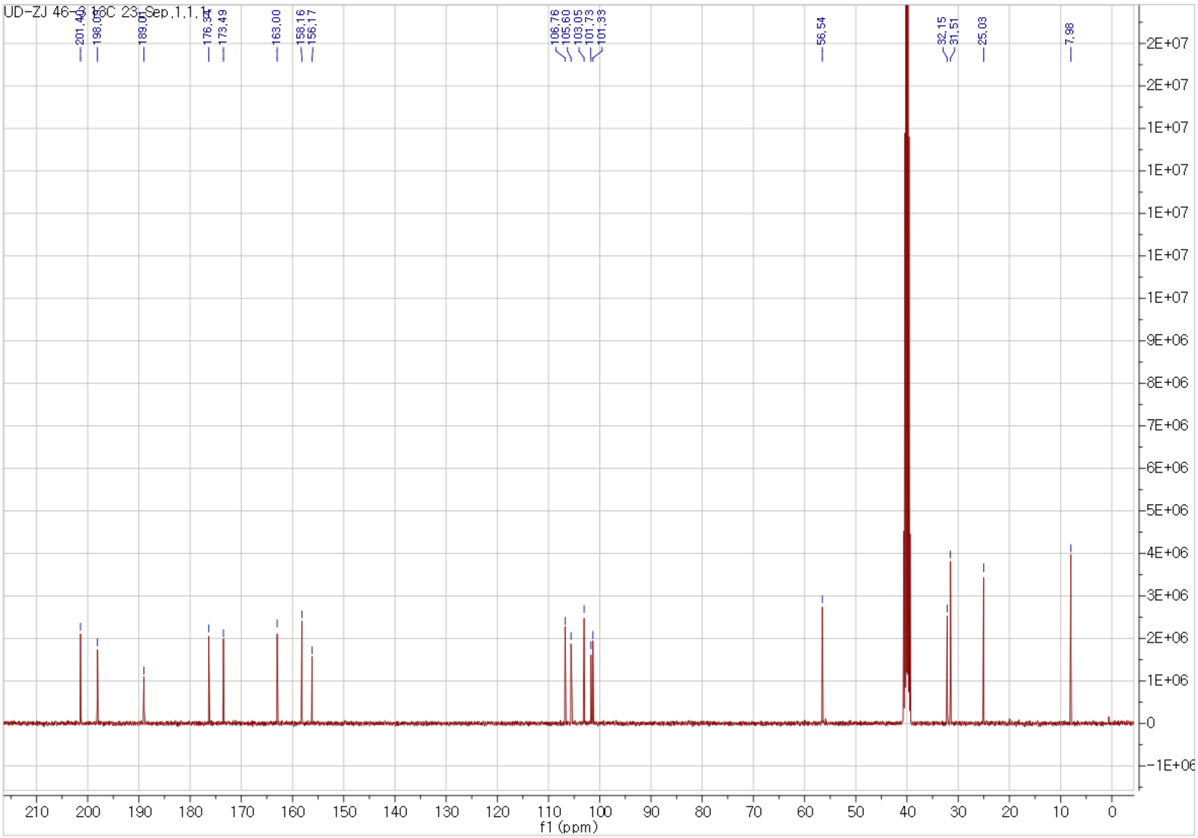

Figure S2. ^{13}C NMR (100 MHz) spectrum of usenamine A in DMSO-d_6.

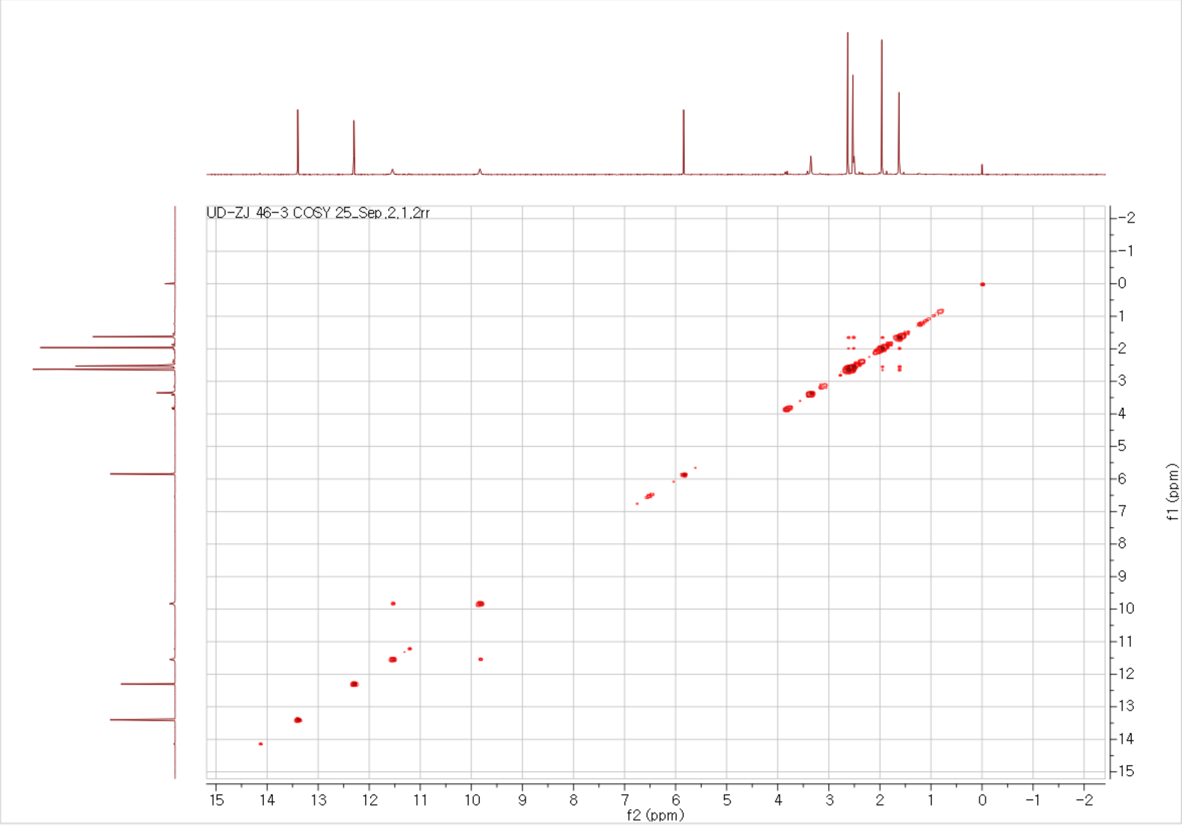

Figure S3. ^{1}H-^{1}H COSY spectrum of usenamine A in DMSO-d_6.

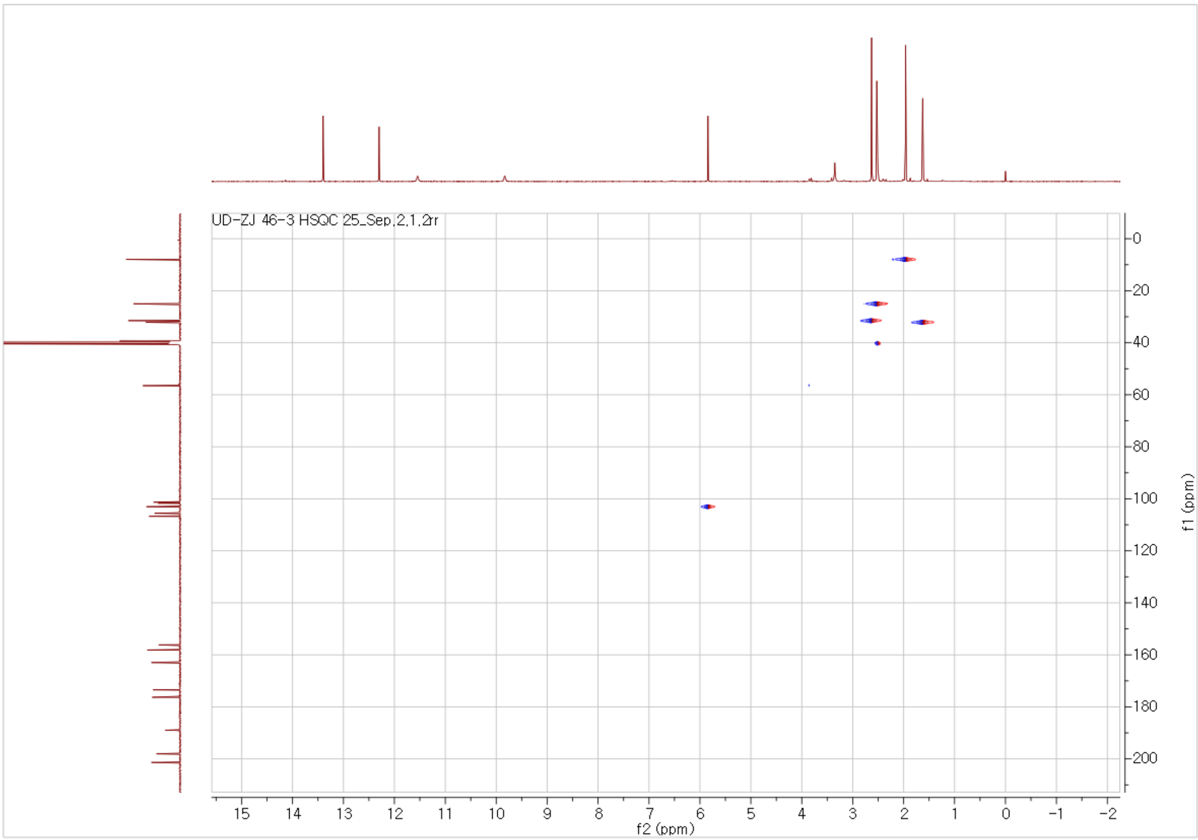

Figure S4. HSQC spectrum of usenamine A in DMSO-d_6.

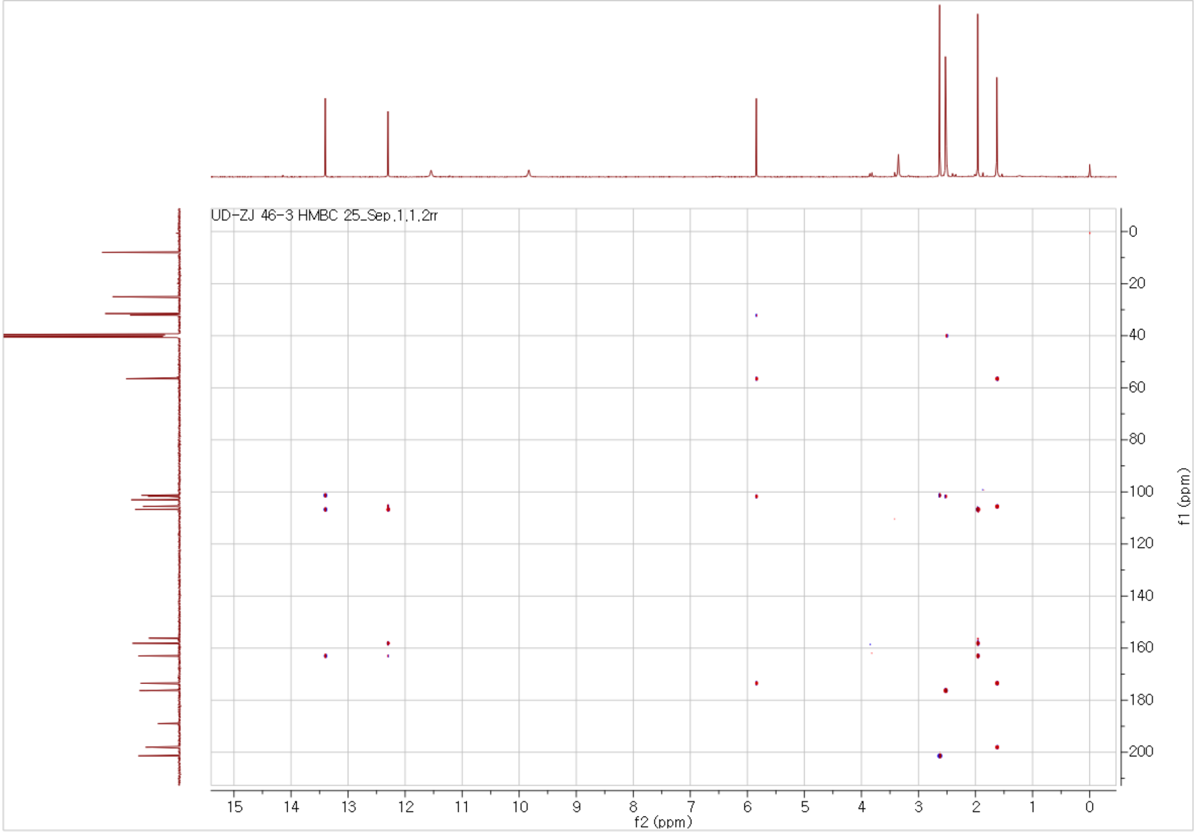

Figure S5. HMBC spectrum of usenamine A in DMSO-*d*6.

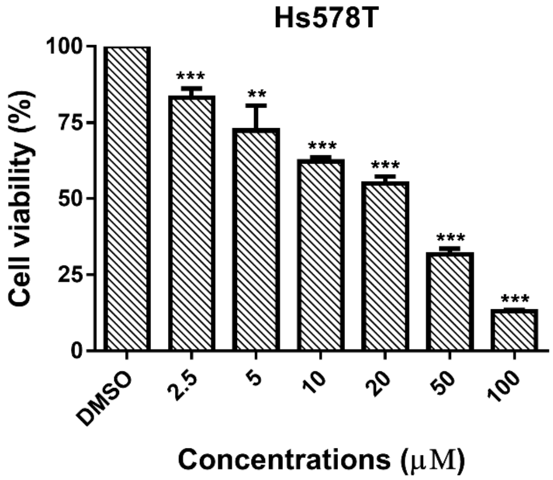

Figure S6. Effect of usenamine A on the viability of triple-negative breast cancer cells. (A) The triple-negative breast cancer cell line, Hs578T, was treated with various concentrations of usenamine A. Cell viability was determined using the MTS assay 48 h after treatment with usenamine A.

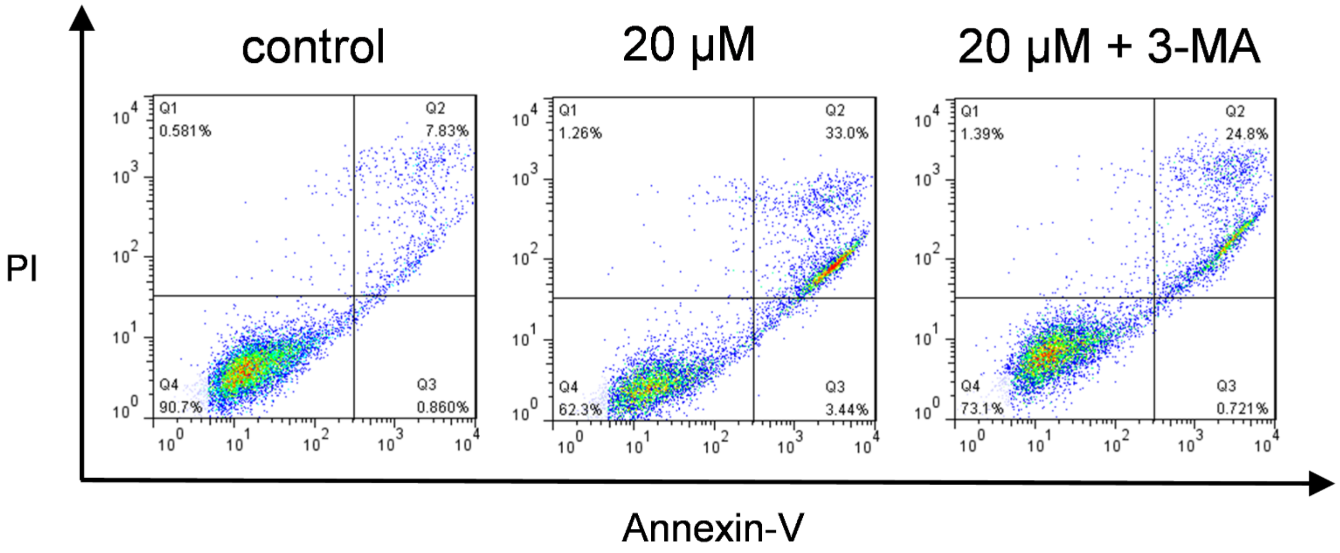

Figure S7. Apoptosis analyses of MDA-MB-231 cells treated with usenamine A and 3-MA. Apoptosis was determined by flow cytometry analysis. The cells were treated with 5 mM 3-Methyladenine (3-MA) for 4 hours then treated with usenamine A (20 μM) for 48 hours.

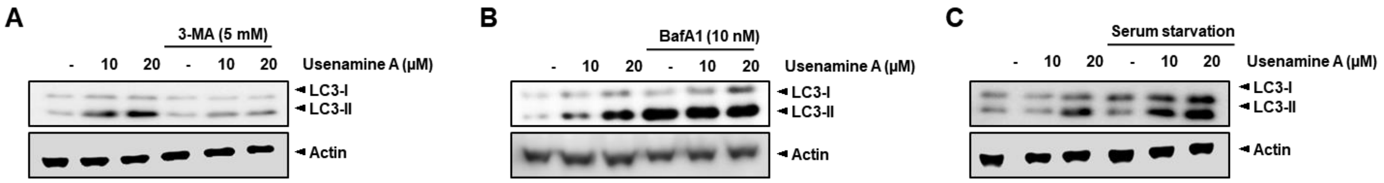

Figure S8. Representative images of immunoblot analysis results of LC3B obtained from autophagy flux assays under (A) 3-MA, (B) BafA1, or (C) serum starvation. The cells were pre-treated with 5 mM 3-MA or 10 nM Bafilomycin A1 (BfaA1) or serum starvation (without FBS) for 4 hours then treated with Usenamine A for 24 hours. .
